# Supplementary material for: A metal ion-dependent mechanism of RAD51 nucleoprotein filament disassembly
Source: iScience. 2023 Apr 25;26(5):106689. doi: 10.1016/j.isci.2023.106689 (PMC10192527; doi:10.1016/j.isci.2023.106689)
Supplement: Document S1. Figures S1–S5 and Tables S1 [file mmc1.pdf]

iScience, Volume *26*

Supplemental information

A metal ion-dependent mechanism of RAD51 nucleoprotein filament disassembly

Robert Appleby, Daniel Bollschweiler, Dimitri Y. Chirgadze, Luay Joudeh, and Luca Pellegrini

Table S1. CryoEM data collection and real-space refinement (related to Figures 1-3).

Data Collection

	**Pre-synaptic**		**Post-synaptic**		**ADP-bound**	
Microscope	Titan Krios					
Voltage (keV)	300					
Detector	Falcon III		K3 (Gatan)			
Collection mode	Integration		Counting			
Magnification	59,000x		130,000x			
Defocus range (μm)	-3.0 to -1.5 (0.3)		-2.5 to -0.9 (0.2)			
Num. movies	1339		3851		9214	
Frames/movie	38		40		50	
Pixel size (Å/pixel)	1.43		0.652 (super-resolution 0.326)			
Electron dose (e$^-$/Å^2/s)	33		47.22		56.36	
Exposure (s)	2		1.61		1.34	
Picked particles	850,217		1,051,037		1,527,085	
Final particles	723,142		543,102		40,481	
Processing method	Helical reconstruction		Single particle		Helical reconstruction	
Map resolution:	masked	unmasked	masked	unmasked	masked	unmasked
d_{FSC} half maps, 0.143	3.7	3.5	2.8	3.0	4.2	4.5
d_{FSC} model, 0.143	3.7	3.7	2.6	2.7	3.7	3.9
Helical twist (°)	56.7		-		53.1	
Helical rise (Å)	16.6		-		18.6	

Real-space refinement

	Pre-synaptic	Post-synaptic	ADP-bound
Composition:			
Non-H atoms	22331	15574	16982
Residues:			
Protein	2790	1866	2191
Nucleotide	30	40	-
Water	-	120	-
Ligands:			
ATP	9	6	
ADP			7
Ca^{2+}	18	12	7
Correlation coefficients:[1]			
CC, mask	0.86	0.83	0.69
CC, peaks	0.66	0.66	0.52
CC, volume	0.85	0.78	0.68
<CC>, ligands	0.86	0.70	0.65
Bonds (rmsd):			
Length(Å)	0.003	0.012	0.005
Angles (°)	0.542	0.643	0.601
MolProbity score[2]	1.42	1.94	2.25
Clash score	7.62	16.40	15.99
Ramachandran plot (%):			
Outliers	0.00	0.00	1.29
Allowed	1.96	2.93	6.47
Favoured	98.04	97.07	92.23
Rotamer outliers (%)	0.00	1.20	1.20
Cb outliers (%)	0.00	0.00	0.00
ADP (B-factors), mean:			
Protein	67.36	27.54	36.07
Nucleotide	58.40	79.69	-
Ligand	53.90	20.34	42.73
Water	-	31.05	-

1. Afonine PV, Klaholz BP, Moriarty NW, Poon BK, Sobolev OV, Terwilliger TC, Adams PD, Urzhumtsev A. New tools for the analysis and validation of cryo-EM maps and atomic models. Acta Crystallographica Section D: Structural Biology. 2018;74(9):814–840.

2. Chen VB, Arendall WB, Headd JJ, Keedy DA, Immormino RM, Kapral GJ, Murray LW, Richardson JS, Richardson DC. MolProbity: all-atom structure validation for macromolecular crystallography. Acta Crystallographica Section D: Biological Crystallography. 2010;66(Pt 1):12–21.

SUPPLEMENTARY FIGURE LEGEND

Figure S1. CryoEM data processing for the pre-synaptic RAD51 NPF, related to Figure 1. **A** Representative micrograph of RAD51 pre-synaptic filament particles. **B** Representative 2D average class. **C** 3D classification and refinement. **D** Fourier Shell Correlation between half maps (top) and model to map (bottom). The resolution at 0.143 FSC is indicated by the intersection of the dashed lines.

Figure S2. CryoEM data processing for the post-synaptic RAD51 NPF, related to Figure 2. **A** Representative micrograph of RAD51 post-synaptic filament particles. **B** Representative 2D average classes. **C** 3D classification and refinement. **D** Fourier Shell Correlation between half maps (top) and model to map (bottom). The resolution at 0.143 FSC is indicated by the intersection of the dashed lines.

Figure S3. Biochemical preparation and cryoEM data processing of the ADP-bound RAD51 filament, related to Figure 3. **A** Electrophoretic mobility shift assay of dsDNA in the presence of RAD51 and increasing amounts of ADP (see Methods for details). **B** Representative micrograph of RAD51 post-synaptic filament particles. **C** Representative 2D average classes. **D** Initial 3D model, two rounds of 3D classification and 3D refinement. **E** Fourier Shell Correlation between half maps (top) and model to map (bottom). The resolution at 0.143 FSC is indicated by the intersection of the dashed lines.

Figure S4. L2 loop conformation in the ADP-bound RAD51 filament, related to Figure 4. The L2 segment not modelled as not visible in the cryoEM reconstruction is drawn as yellow dots. **A** Two adjacent RAD51 protomers are shown in light brown and orange. The L2 loop is highlighted in yellow, with residue side chains shown. Residues that are engaged in prominent hydrophobic interactions are labelled. **B** View of the L2 loop conformation that highlights its likely steric clash with the DNA. Colour-coding as in panel A. The inner strand of the dsDNA from the post-synaptic filament is shown as stick model and transparent molecular surface.

Figure S5. Evidence for the presence of a second metal ion at the ATP-binding site in high-resolution cryoEM maps of pre-synaptic human RAD51-AMPPNP-Mg^{2+} (EMD 31158, PDB 7EJC), pre-synaptic human DMC1-AMPPNP-Ca^{2+} (EMD 30311, PDB 7C9C) and post-synaptic yeast DMC1-ATP-Mg^{2+} (EMD 31154, PDB 7EJ7), related to Figures 1 and 2. The position of the density peak attributable to the second metal cation is indicated by an arrow. The ATP and the side chains of important ATP binding-residues are drawn explicitly.

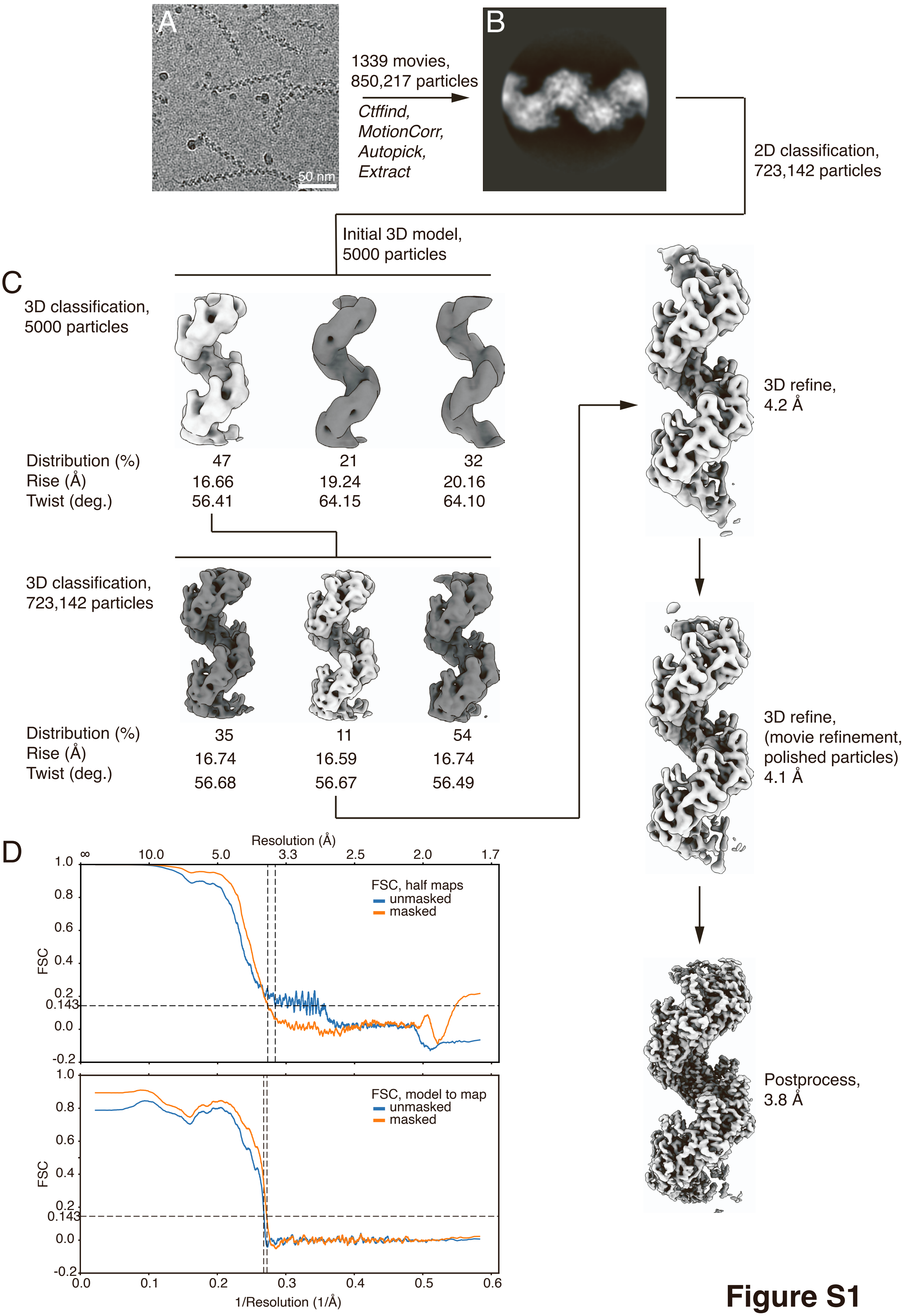
A
50 nm
1339 movies,
850,217 particles
Ctffind,
MotionCorr,
Autopick,
Extract
B
2D classification,
723,142 particles
Initial 3D model,
5000 particles
C
3D classification,
5000 particles
Distribution (%) 47 21 32
Rise (Å) 16.66 19.24 20.16
Twist (deg.) 56.41 64.15 64.10
3D classification,
723,142 particles
Distribution (%) 35 11 54
Rise (Å) 16.74 16.59 16.74
Twist (deg.) 56.68 56.67 56.49
3D refine,
4.2 Å
3D refine,
(movie refinement,
polished particles)
4.1 Å
Postprocess,
3.8 Å
D
Resolution (Å)
∞ 10.0 5.0 3.3 2.5 2.0 1.7
FSC, half maps
unmasked
masked
FSC, model to map
unmasked
masked
FSC
1.0 0.8 0.6 0.4 0.2 0.143 0.0 -0.2
1/Resolution (1/Å)
0.0 0.1 0.2 0.3 0.4 0.5 0.6

Figure S1

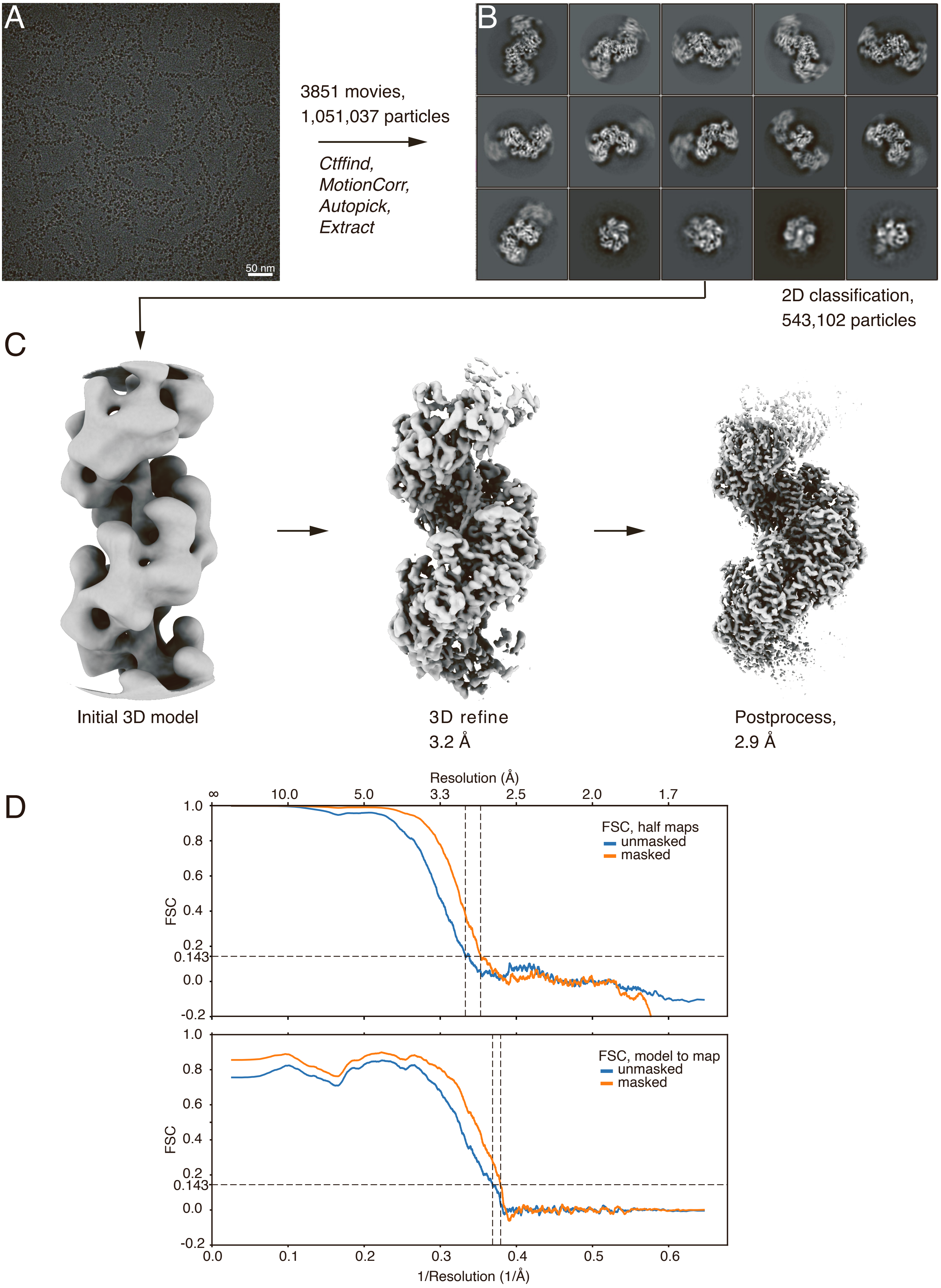
A
50 nm
3851 movies,
1,051,037 particles
Ctffind,
MotionCorr,
Autopick,
Extract
B
2D classification,
543,102 particles
C
Initial 3D model
3D refine
3.2 Å
Postprocess,
2.9 Å
D
Resolution (Å)
∞
10.0
5.0
3.3
2.5
2.0
1.7
FSC, half maps
unmasked
masked
FSC
1.0
0.8
0.6
0.4
0.2
0.143
0.0
-0.2
FSC, model to map
unmasked
masked
0.0
0.1
0.2
0.3
0.4
0.5
0.6
1/Resolution (1/Å)

Figure S2

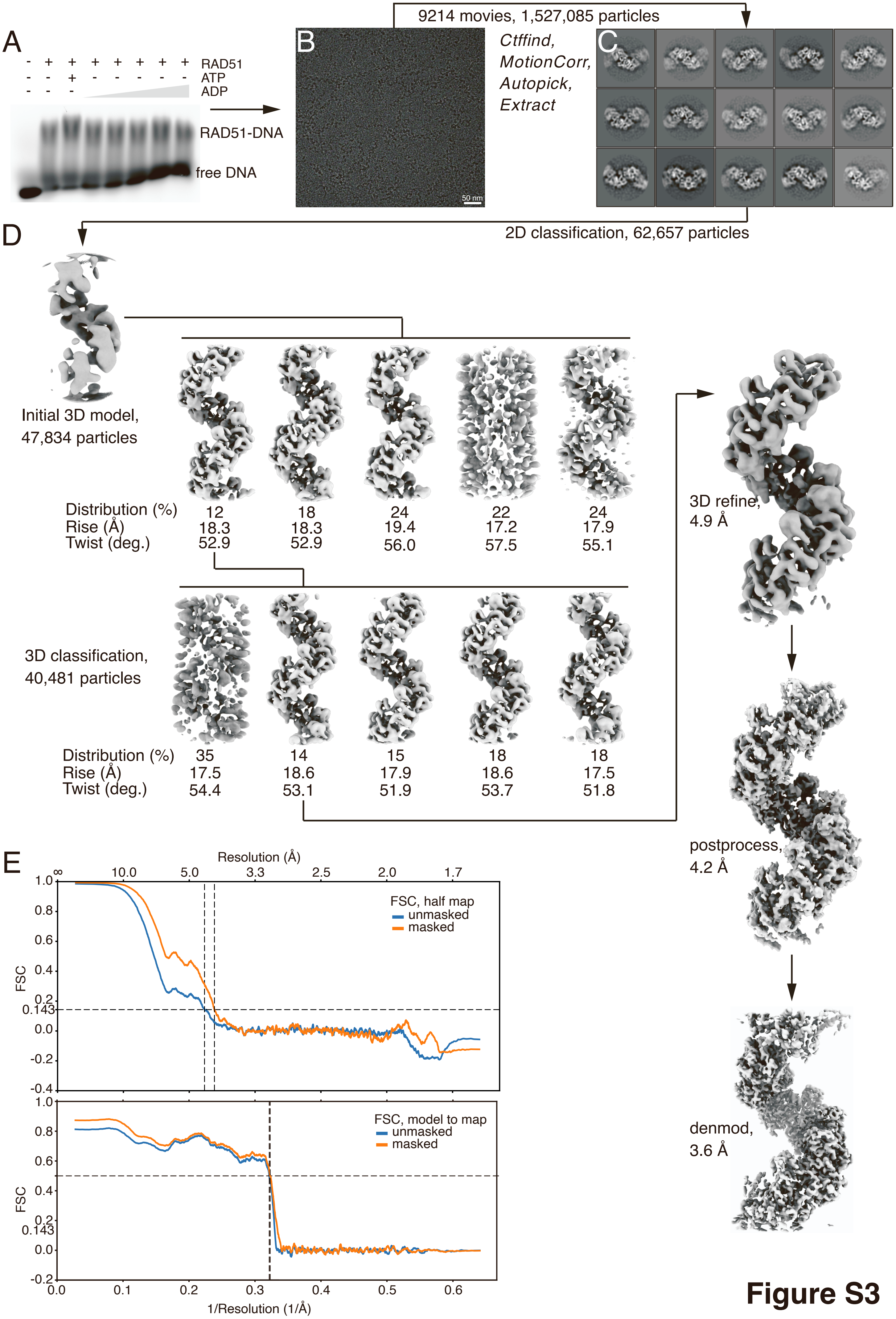
A
RAD51
ATP
ADP
RAD51-DNA
free DNA
B
50 nm
9214 movies, 1,527,085 particles
Ctffind,
MotionCorr,
Autopick,
Extract
C
2D classification, 62,657 particles
D
Initial 3D model,
47,834 particles
Distribution (%) 12 18 24 22 24
Rise (Å) 18.3 18.3 19.4 17.2 17.9
Twist (deg.) 52.9 52.9 56.0 57.5 55.1
3D classification,
40,481 particles
Distribution (%) 35 14 15 18 18
Rise (Å) 17.5 18.6 17.9 18.6 17.5
Twist (deg.) 54.4 53.1 51.9 53.7 51.8
3D refine,
4.9 Å
postprocess,
4.2 Å
denmod,
3.6 Å
E
Resolution (Å)
FSC, half map
unmasked
masked
FSC, model to map
unmasked
masked
FSC
1/Resolution (1/Å)

Figure S3

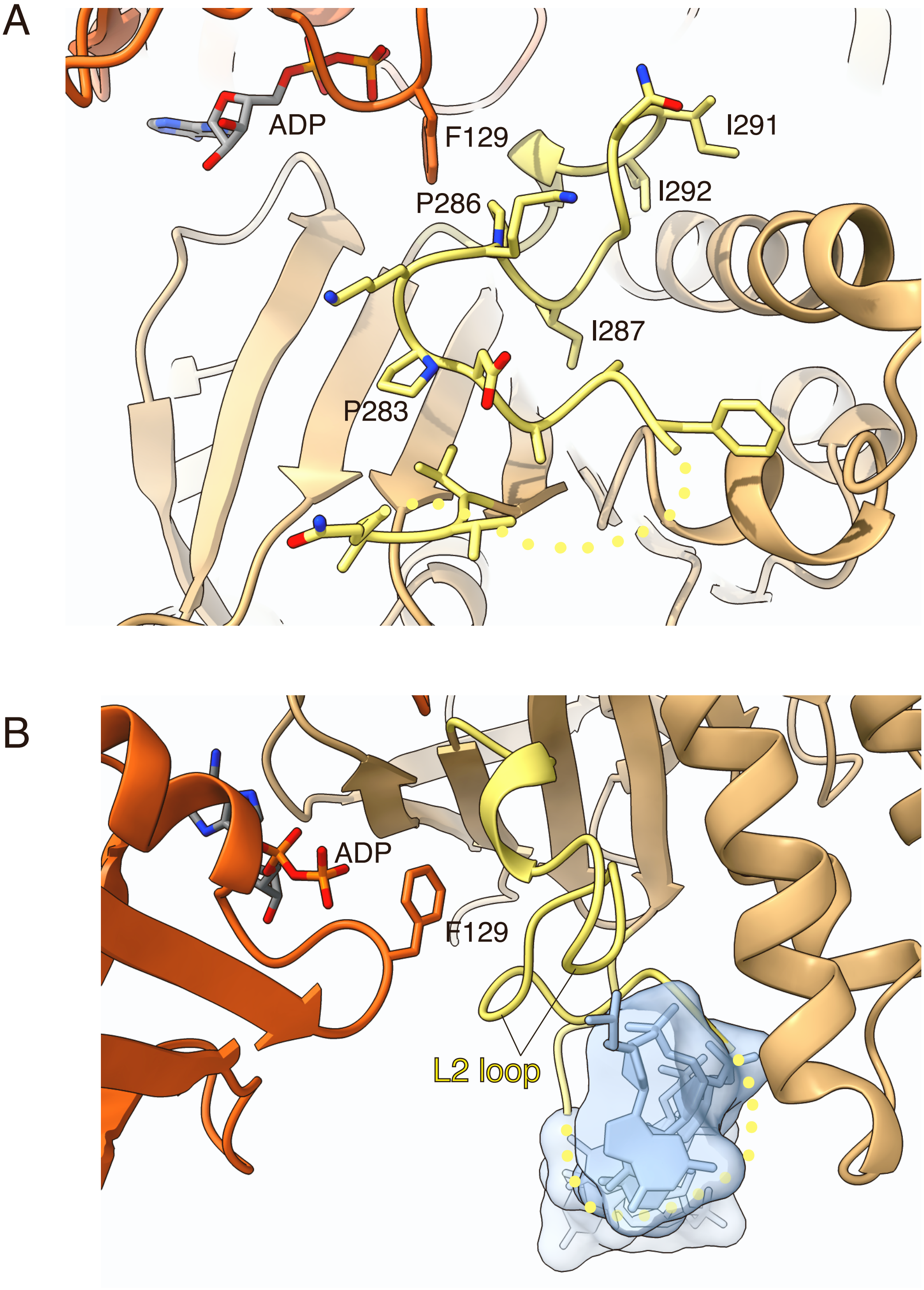

Figure S4

RAD51
Pre-synaptic filament
PDB ID: 7EJC
2.97 Å

DMC1
Pre-synaptic filament
PDB ID: 7C9C
3.33 Å

DMC1
Post-synaptic filament
PDB ID: 7EJ7
3.41 Å

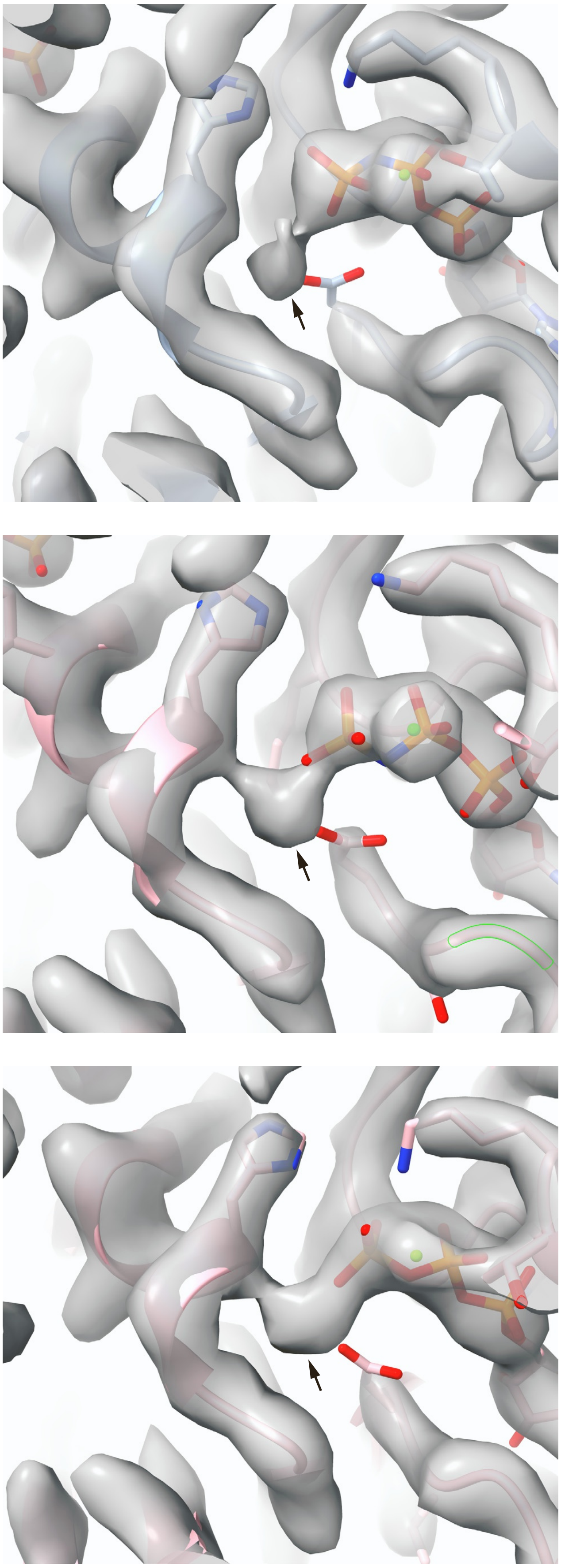

Figure S5
